# Aluminum doped zinc oxide deposited by atomic layer deposition and its applications to micro/nano devices

**DOI:** 10.1038/s41598-020-80880-3

**Published:** 2021-01-13

**Authors:** Nguyen Van Toan, Truong Thi Kim Tuoi, Naoki Inomata, Masaya Toda, Takahito Ono

**Affiliations:** 1 Micro System Integration Center, Tohoku University, Sendai, Japan; 2 Department of Mechanical Systems Engineering, Tohoku University, Sendai, Japan

**Keywords:** Circulation, Physiology, Cardiology

## Abstract

This work reports investigation on the deposition and evaluation of an aluminum-doped zinc oxide (AZO) thin film and its novel applications to micro- and nano-devices. The AZO thin film is deposited successfully by atomic layer deposition (ALD). 50 nm-thick AZO film with high uniformity is checked by scanning electron microscopy. The element composition of the deposited film with various aluminum dopant concentration is analyzed by energy-dispersive X-ray spectroscopy. In addition, a polycrystalline feature of the deposited film is confirmed by selected area electron diffraction and high-resolution transmission electron microscopy. The lowest sheet resistance of the deposited AZO film is found at 0.7 kΩ/□ with the aluminum dopant concentration at 5 at.%. A novel method employed the ALD in combination with the sacrificial silicon structures is proposed which opens the way to create the ultra-high aspect ratio AZO structures. Moreover, based on this finding, three kinds of micro- and nano-devices employing the deposited AZO thin film have been proposed and demonstrated. Firstly, nanowalled micro-hollows with an aspect ratio of 300 and a height of 15 µm are successfully produced . Secondly, micro- and nano-fluidics, including a hollow fluidic channel with a nanowall structure as a resonator and a fluidic capillary window as an optical modulator is proposed and demonstrated. Lastly, nanomechanical resonators consisting of a bridged nanobeam structure and a vertical nanomechanical capacitive resonator are fabricated and evaluated.

## Introduction

High aspect ratio micro- and nano-structures are key factors to achieve high performances of the various micro- and nano-devices. For example, the high aspect ratio micro- and nano-channels are needed for micro- and nano-fluidics to measure the mass of a single cell or single micro- and nano-particle^1^, for liquid energy harvesting^2^, and thermal transpiration pump^3^. The high aspect ratio micro- and nano-needles are required to precise injector for cells^4^. The high aspect ratio micro- and nano-mechanical structures are typically used to improve the sensitivity of micro- and nano-devices^5^. The high aspect ratio is simply defined as the ratio between the vertical dimension and lateral dimension. Dry etching processes such as inductively coupled plasma reactive ion etching (ICP-RIE) using a well-established Bosch process have been widely used to form the high aspect ratio of the etched silicon structures^6–8^; however, the achievable aspect ratio is limited by anisotropy and etch selectivity. Recently, to create the high aspect ratio silicon structures, the metal-assisted chemical etching (MACE) method is focused and demonstrated^9–13^. Although ultra-high aspect ratio of the etched structures has been achieved by MACE, the patterning of large-size structures still faces problems owing to the non-uniform etching rate and catalyst metal degradation^14^. Therefore, the fabrication for micro- and nano-devices requiring large and narrow patterning areas becomes a challenge for MACE. Consequently, a novel way to create high aspect ratio micro- and nano-structures is still required for micro- and nano-systems.

Zinc oxide (ZnO) is an attractive semiconductor material because of its wide bandgap (3.3 eV at 300 K), high Young’s modulus (150–240 GPa for thin films), high thermal and chemical stability, and compatibility with micro- and nano-fabrication techniques. Also, the electrical conductance of the ZnO can be significantly improved by aluminum doping^15^. Especially, ZnO is a promising candidate to replace silicon for the micro/nano electromechanical systems (MEMS/NEMS)^16^. Several techniques have been implemented to synthesize ZnO films, including radio-frequency (RF) magnetron sputtering^17,18^, direct current (DC) sputtering^19^, chemical vapor deposition (CVD)^20^, pulsed laser deposition (PLD)^21^ and atomic layer deposition (ALD)^22–24^. ZnO properties, including optical, electrical, and mechanical characteristics, have been investigated in several works^18–24^. Among the above-mentioned techniques, the ALD method allows the deposition of uniform and conformal ZnO thin films with atomic-level control of thickness on a three-dimensional surface^24^. ZnO material has been employed for several applications, including electronics^25–27^, optoelectronics^28^, piezoelectric transducers^30,31^, and sensors^32–34^. All aforementioned devices employ the ZnO thin film deposition; however, for thick film deposition such as several tens of micrometers, there are no investigations due to the low deposition rate of the current technologies. ZnO structures could be patterned by both wet and dry etching techniques, which pose advantages as well as disadvantages. A high etching rate could be achieved by a wet etching technique, but the lateral etching is its problem. In contrast, dry etching techniques including reactive ion etching (RIE) and ion beam milling technique can apply to precise patterning; nevertheless, the achievable aspect ratio is limited by photoresist resolution and etch selectivity. In such situations, the methods to create high aspect ratio ZnO structures for micro- and nano-devices need to be addressed.

A novel process of a combination of the selective epitaxial growth (SEG) and epitaxial lateral overgrowth (ELO) has been proposed to create high aspect ratio structures^35^ and three-dimensional devices^36^. The process involves: firstly, a sacrificial layer deposition (typically *SiO*_2_) is deposited and patterned which is followed by the deposition and patterning of the silicon or polysilicon. Finally, the sacrificial layer is removed to leave the freestanding structures. Although SEG and ELO have been demonstrated successfully, these techniques are complex. Besides, high-temperature process treatment is required which may affect other materials and structures on the wafer. To achieve high quality deposited films, several parameters, including temperature, pressure, and gas flows, need to be optimized. Also, defect density, crystal lattice, film stress, and large mismatch in the thermal expansion coefficients need to be addressed. Typically, the sacrificial oxide layer is removed by the wet etching method which results in the sticking issue for the high aspect ratio structures. Moreover, the aspect ratio for ELO growth on Si is about 1:1 and the low deposition rate is its characteristic of around 0.15 µm/min, as given in^37^. This implies that, for large area coverage of oxide by silicon, the deposition process takes for long times, which might not be economically feasible. In this work, the use of the ALD in combination with the sacrificial silicon structures is proposed to create the high aspect ratio AZO structures not only in lateral directions but also in vertical directions. The aluminum-doped zinc oxide (AZO) thin film is studied by the ALD. Moreover, three kinds of micro- and nano-devices with high aspect ratio structures, including micro-hollows, micro- and nano-fluidics, and nanomechanical resonators have been proposed, fabricated, and evaluated.

## Deposition and evaluation of AZO thin film

### Atomic layer deposition process

The ALD process poses a precise deposition, high uniformity, and conformal thin film onto complex three-dimensional topographies. Herein, an AZO thin film is deposited by employing trimethylaluminum (TMA), diethyl zinc (DEZ), and deionized (DI) water with a hot-wall atomic layer deposition system (Picosun, Finland). The deposition chamber temperature is 200 °C. DEZ is used as a precursor while DI water is employed as an oxidant source for forming the ZnO. After 20 cycles of forming ZnO layers, a single cycle of aluminum doping (TMA and DI water) is conducted. The deposition process is repeated until achieving the expected thickness. A similar description of the ALD process for the AZO thin film could be also found at^38^. The AZO is coated on the entire surface of various substrates, including glass substrates, silicon substrates, and the patterned silicon substrate for the material evaluation and fabrication of micro- and nano-devices which will be presented in late sections.

### Material evaluation

Morphology is studied by a scanning electron microscopy (SEM) and the element composition of the electrodeposited film is analyzed using energy-dispersive X-ray spectroscopy (EDX). Transmission electron microscopy (TEM) and selected area electron diffraction (SAED) are employed to evaluate the nanostructures of the deposited films. The electrical property of the deposited films is evaluated by a four-point probe resistance measurement.

**Figure 1:**
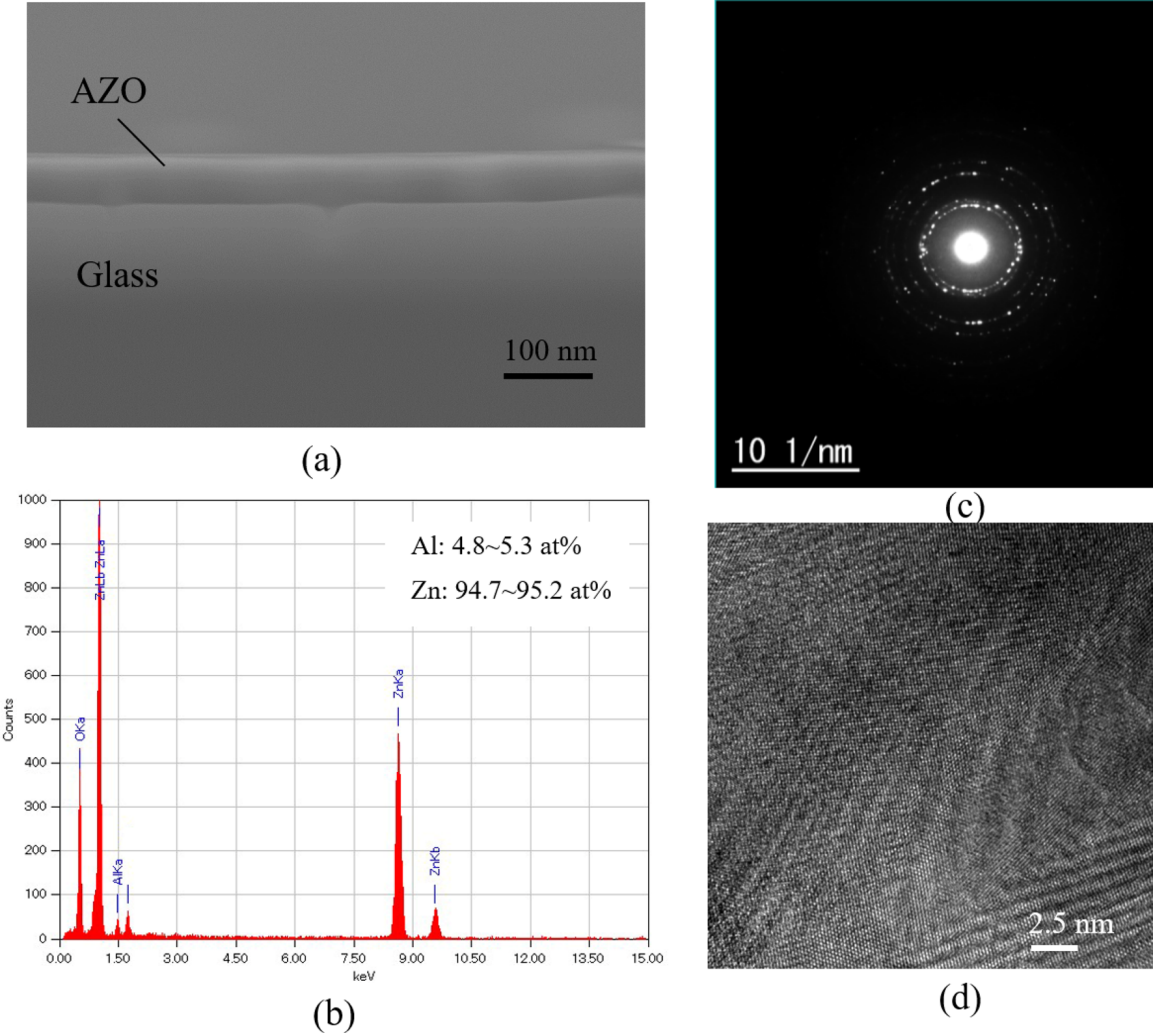
Morphology analysis. (**a**) Cross-sectional SEM image of the deposited film. (**b**) Element composition of the deposited film was analyzed by EDX. (**c**) Selected area electron diffraction of the film. (**d**) Transmission electron microscopy image of the film.

The AZO is successfully deposited on a 300 µm-thick glass substrate for the evaluations of the material properties. The deposition rate of the AZO film is observed at 0.15 nm per cycle and approximately 50 nm-thick AZO is grown successfully, as shown in Fig. 1a. The deposition rates in cases with and without aluminum doping are almost the same. The deposited film shows high uniformity. The element composition on the surface of the deposited films is analyzed by EDX, as shown in Fig. 1b. In this substrate, the composition of the aluminum is found to be 4.8 ~ 5.3 at.% while that of Zn is 94.7 ~ 95.2 at.%. Furthermore, the structure information of the AZO is investigated by the selected area electron diffraction (SAED). As can be seen in Fig. 1c, the SAED pattern shows a circular diffraction pattern and those cycle rings are composed of bright spots. This means that the AZO thin film deposited by ALD is polycrystalline. This evidence is also confirmed by a high-resolution TEM, as given in Fig. 1d.

**Figure 2:**
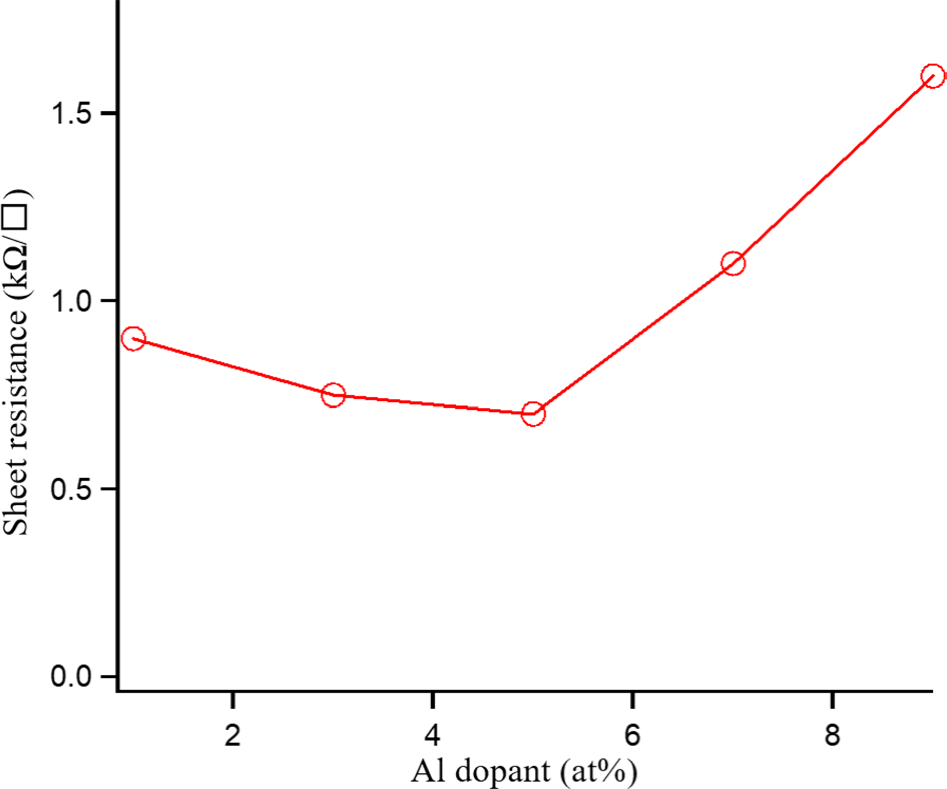
Dependency of the sheet resistance of AZO film by ALD on aluminum dopant concentration.

The electrical property of the AZO thin films with various aluminum dopant concentrations is evaluated, as shown in Fig. 2. In this evaluation, each point is measured five times at different areas on a 2 × 2 cm^2^ wafer and the result of each point is recorded by the average value. The sheet resistance of the deposited AZO film as a function of the Al:Zn atomic ratio has been found. Figure 2 shows that the sheet resistance of the deposited films decreases from 0.9 kΩ/□ to 0.7 kΩ/□ with increasing the aluminum dopant concentration from 1 at.% to 5 at.%. Then, it increases for the deposited films with higher aluminum contents. This phenomenon could be explained as follows. The decrease of the sheet resistance as increasing the dopant concentration from 1 at.% to 5 at.% can be attributed to the increase of the grain size of the AZO film which could help to reduce the grain-boundary scattering (increasing carrier concentration). After reaching the lowest sheet resistance at the aluminum dopant of 5 at.%, the sheet resistance increases because of the decrease of the electron mobility which may come from the intrinsic defects such as imperfect crystalline lattice and vacancy defects. A similar trend of the sheet resistance of the deposited AZO thin film depending on aluminum doping concentration could be found in other literatures^39,40^.

Besides the metal doping method to enhance the electrical property of ZnO film, the surface passivation effect by fluorine plasma treatment could also help to improve the electrical conductivity of ZnO film, as presented in^39^. Fluorine ions substitute oxygen and/or oxygen vacancies in the ZnO atomic structure due to a similar ionic radius of fluorine ions and oxygen ions during the plasma treatment. These replacements could increase the carrier mobility of ZnO:F film, as presented in^41,42^. Thus, the electrical conductivity of the ZnO film could be enhanced by the *SF*_6_ plasma treatment. Herein, the passivation effect of *SF*_6_ plasma on 50 nm-thick AZO film deposited on the glass substrate by the ALD is examined. The experimental result shows that the sheet resistance of AZO film is almost no change with and without the plasma treatment. Compared to previous work^41^, the deposited AZO film by the ALD method is denser than that of the ZnO film by the sol–gel method. Thus, the diffusion of fluorine ions is limited from the film’s surface only and therefore almost no changes in the electrical conductivity of AZO film have been found under the plasma treatment.

## Typical applications

### Micro-hollows with nanowall structure

Micro-hollow structures pose great potential for biomedical applications^43,44^. For instance, by employing micro-hollows as drug containers, tiny amount and accurate drugs could be delivered to the target. This leads to control of the drug dose and its release, thereby achieving optimal therapeutic effect and meanwhile mitigating toxicity. Silicon hollows with a nanowall of 100 nm thickness with a height-to-width aspect ratio of 16 are demonstrated in^45^. A Si nanobarrel structure with 6.7 nm thick nanowall corresponding to an aspect of 50 is reported in^46^, but its height is 335 nm. In general, the fabrication process of the high aspect ratio micro-hollow structures is complicated as well as fabrication cost is high. Herein, a simple and cost-effective fabrication method for the high aspect ratio AZO hollow structures is proposed and investigated.

**Figure 3:**
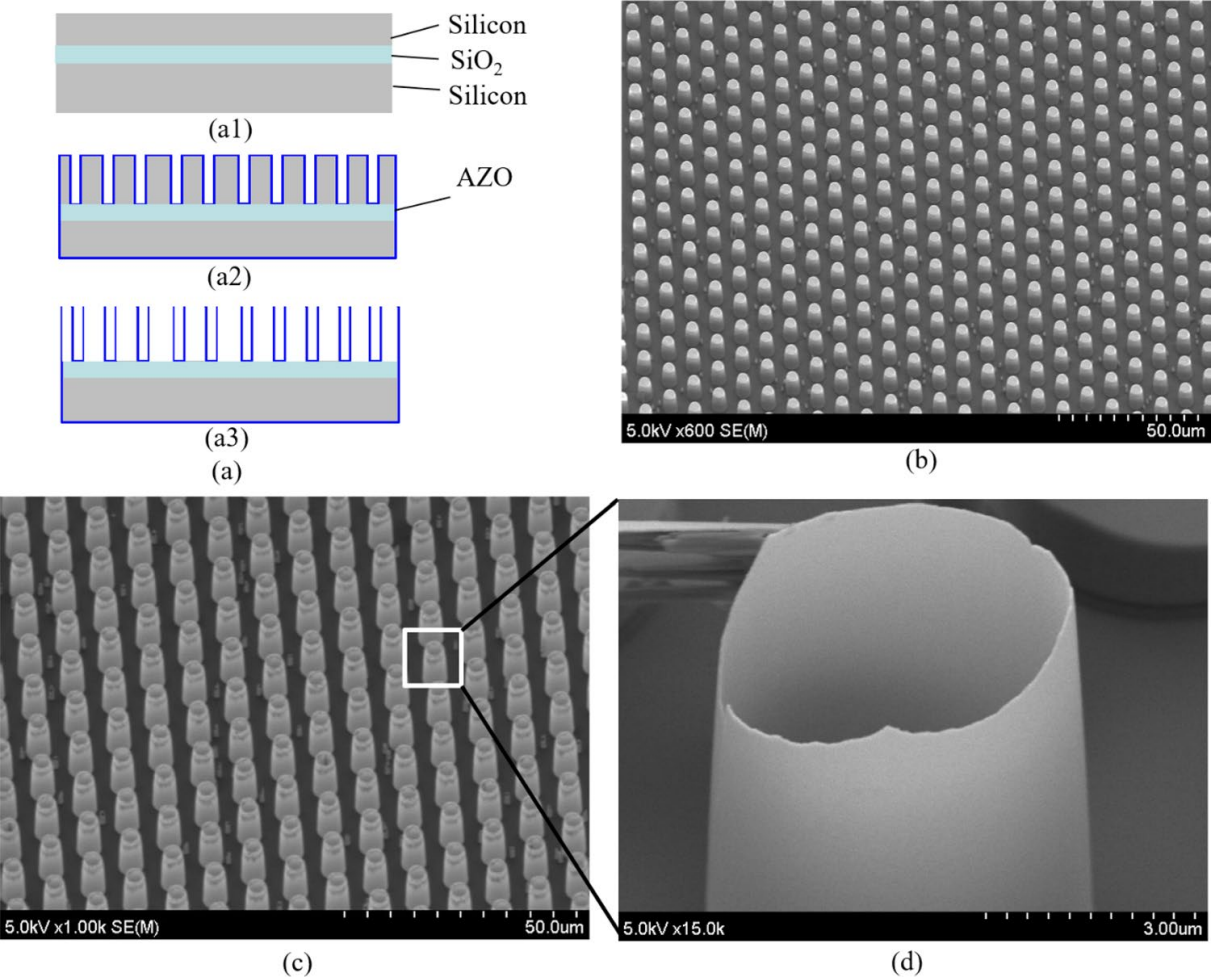
Micro-hollows with nanowall structure. (**a**) Fabrication process. (**a1**) SOI wafer. (**a2**) Deep RIE and ALD. (**a3**) Silicon etching. (**b**) Micro-pillar silicon structures. (**c**) AZO hollows. (**d**) Close-up image of AZO hollow with nanowall structure.

The fabrication of micro-hollows with the nanowall structure is carried out in a combination of the ALD and deep RIE, as shown in Fig. 3a. The process begins from a silicon on insulator (SOI) wafer with a 15 µm-thick device layer, 1 µm-thick oxide layer, and 300 µm-thick silicon handling layer (Fig. 3a1). After conventional cleaning processes, the silicon wafer is patterned to form silicon pillars with a diameter and height of 5 µm and 15 µm, respectively, by the deep RIE employed Bosch process using a photoresist as a protected mask. Next, a 5% aluminum-doped ZnO layer with a thickness of 50 nm is deposited on the patterned silicon wafer by the ALD (Fig. 3a2). The ALD process is described in the previous section. The AZO film on the top of the silicon pillar’s surface is sequentially etched out by ion beam milling. Finally, silicon pillars are removed by *SF*_6_ plasma etching. The etched results show that silicon can be removed selectively without any influence on the deposited AZO thin film. The possible reasons are due to the high etching selectivity of zinc and aluminum in AZO film to fluoride gas. During the *SF*_6_ plasma process, fluorides of zinc and aluminum are formed on the surface of AZO film which is non-volatile very stable compounds. Similar techniques have been reported previously in the fabrication of the high aspect ratio Al_2_O_3_ and TiO_2_ trenches^47,48^. Micro-pillar silicon structures formed by deep RIE are shown in Fig. 3b. The SEM images of the fabricated nanowall micro-hollows with an aspect ratio of 300 and their height of 15 µm are shown in Fig. 3c, d. Much higher aspect ratio nanowall structures are possibly produced by utilizing higher aspect ratio silicon molds. Thus, the use of ALD in a combination with sacrificial silicon structures poses a novel way to create large scale high aspect ratio AZO structures which could be helpful for many different applications in biomedical engineering and nanomedicine.

## Micro- and nano-fluids

In this section, two kinds of micro- and nano-fluidic devices including a hollow fluidic channel with nanowall structure as a resonator and a fluidic capillary window as an optical modulator are proposed and investigated.

Firstly, the hollow fluidic channel as a resonator has been attention due to its high sensitivity for particles suspended in liquid^1,49–51^. The mass, density, and volume of a single biological cell or single micro- and nano-particle could be quantitatively measured, which is based on changing the device resonant frequency. To further improve the performance of the hollow fluidic resonators, considerable effort is made to reduce their physical sizes such as the wall thickness. Although hollow fluidic resonators are successfully produced by the silicon fusion bonding method^49^ and silicon-on-nothing (SON) technique^50^, those fabrication processes are complex and take time. Herein, the use of ALD in combination with the sacrificial silicon etching process is proposed to create the hollow fluidic channel with the nanowall structures. The method is based on the ALD deposition on the patterned silicon structures with a sub-sequential removal of the patterned silicon structures by *SF*_6_ plasma etching.

**Figure 4:**
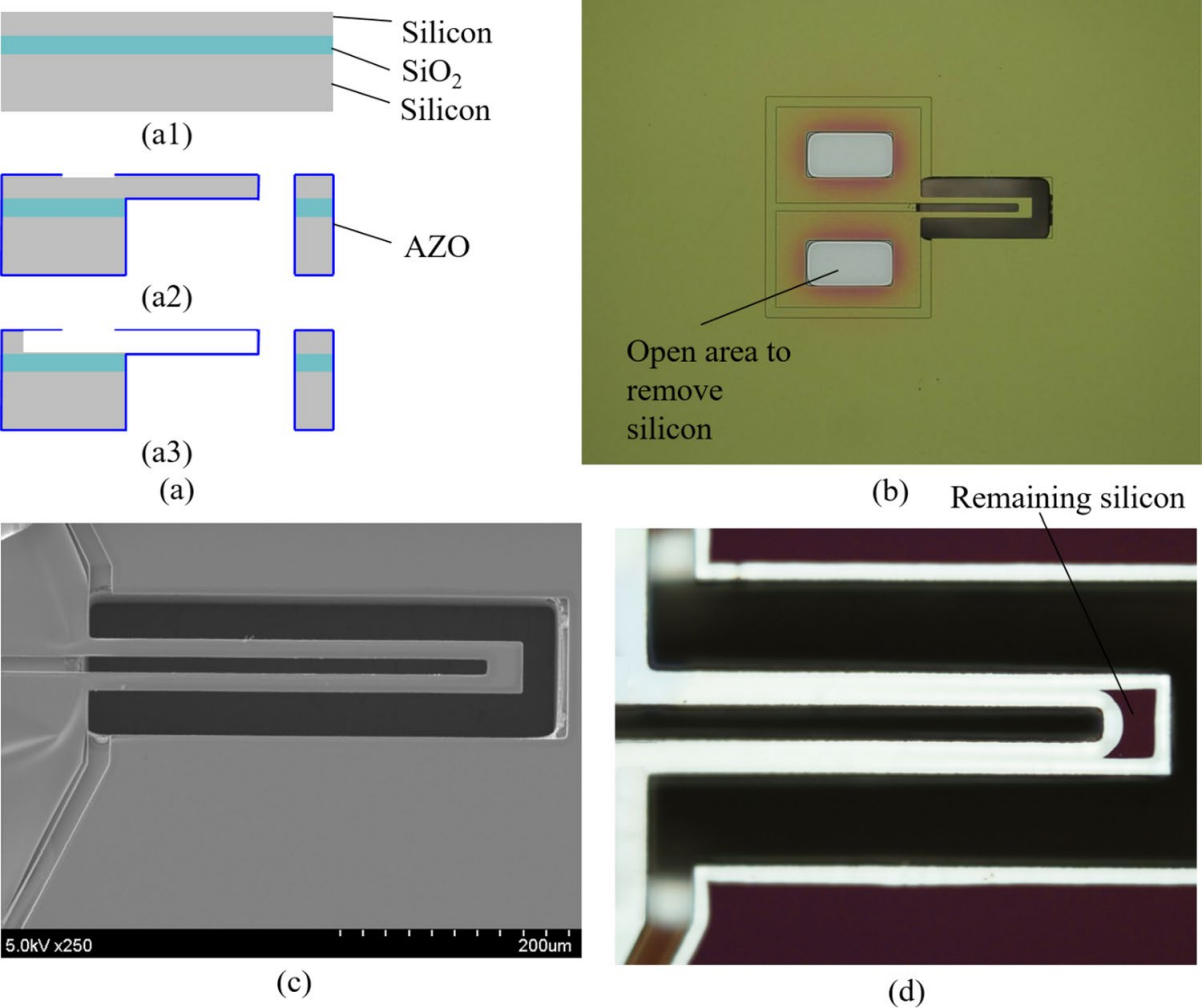
Hollow fluidic channel. (**a**) Fabrication process. (**a1**) SOI wafer. (**a2**) Silicon cantilever with a patterned coating AZO. (**a3**) Removal of silicon structure. (**b**) Optical image of a silicon cantilever with a patterned coating AZO. (**c**) SEM image of the completely fabricated structure. (**d**) Optical image of the completely fabricated structure.

Figure 4a shows the fabrication process for the AZO hollow fluidic channel. It starts with an SOI wafer (same as mentioned above), as shown in Fig. 4a1. Using conventional micro-fabrication technologies including photolithography and deep RIE, the micro-cantilever is produced. More information about the similar fabrication process could be found in our previous works^52,53^. Next, a 50 nm-thick AZO layer is deposited on the patterned silicon structures by the ALD, and the AZO layer is partly etched via a stencil mask by ion beam milling technique, as shown in Fig. 4a2. Finally, the cantilever silicon structure as a sacrificial layer is removed by *SF*_6_ plasma etching (Fig. 4a3). Figure 4b is an optical image of a silicon cantilever with a patterned AZO layer which corresponds to step in Fig. 4a2. The completed hollow fluidic structure is shown in Fig. 4c. Although a small part of silicon at the tip of the cantilever remains (Fig. 4d), both sides of the channel are connected. This remaining silicon is possibly removed by extending the etching time of the deep RIE process. The hollow fluidic channel with the nanowall structure has been fabricated successfully; however, liquid injection to the micro-channel is facing a problem because of the small size of the fabricated device. Thus, an assembling method for liquid injection need to be established. More consideration to solve this problem is being conducted and will be presented in our further investigations.

Secondly, the fluidic capillary window as the optical modulator is investigated and demonstrated. The fluidic capillary window is operated by utilizing the electrowetting phenomenon which is employed for various applications including displays^54,55^, optics^56^, energy harvesting^57^, and lab-on-chip^58^. The contact angle of the liquid–solid surface could be controlled when an electric field is created by applying a potential. The merit of electrowetting is the ability to electrically manipulate liquid droplets without movable mechanical parts. Thus, the power consumption of systems is small. Generally, the electrowetting is demonstrated on a dielectric film. The actuated voltage requirement needs to be lowered which can toward practical applications. Some efforts to reduce the actuated voltage including a decreasing film thickness^59^ and increasing film dielectric constant^60^ have been demonstrated. However, several electrowetting systems still require high voltage supplies^61^ while most electric devices operate at a much lower voltage. Therefore, an integration between electrowetting and circuits becomes a challenge. Herein, the fluidic capillary window as the optical modulator is proposed and demonstrated which may replace for microlens array in the current digital cameras. The light transmission could be modulated by a liquid level in capillaries, and the transmission is controlled by moving the liquid using reversible electrowetting.

The device concept of the optical modulator has been reported in our previous works^60–64^ which is briefly summarized, as follows. The light transmission through the optical window is controlled by a liquid movement in the SiO_2_ capillaries^62,63^, pillars^64,65^, or nano-trenches^66^. When the electric field is applied to the structure, the liquid moves into the SiO_2_ patterning. Thus, it makes the structure becomes like a transparent solid structure (reflective index of liquid and SiO_2_ materials should be similar); therefore, high light transmittance can be achieved. In the case without liquid in the SiO_2_ pattern the light transmittance of the device is lower than that with liquid because of light reflection and scattering by capillary walls. By controlling the liquid level inside the capillaries via voltage supplies, the light-optical modulation is possibly controlled. Although the aforementioned devices^62–66^ pose excellent optical characteristics, a complete description of the device working concept based on the electrowetting has not been demonstrated yet owing to requirements of metal deposition, dielectric and hydrophobic coating. Herein, we extend our previous works and perform a more systematic study of this phenomenon. The conductive AZO metal layer is successfully deposited by the ALD, and the hydrophobic coating is investigated by a dip coating machine. Also, the fabrication process is improved to enhance the mechanical strength of the optical modulator device compared to previously reported devices^60–64^.

**Figure 5:**
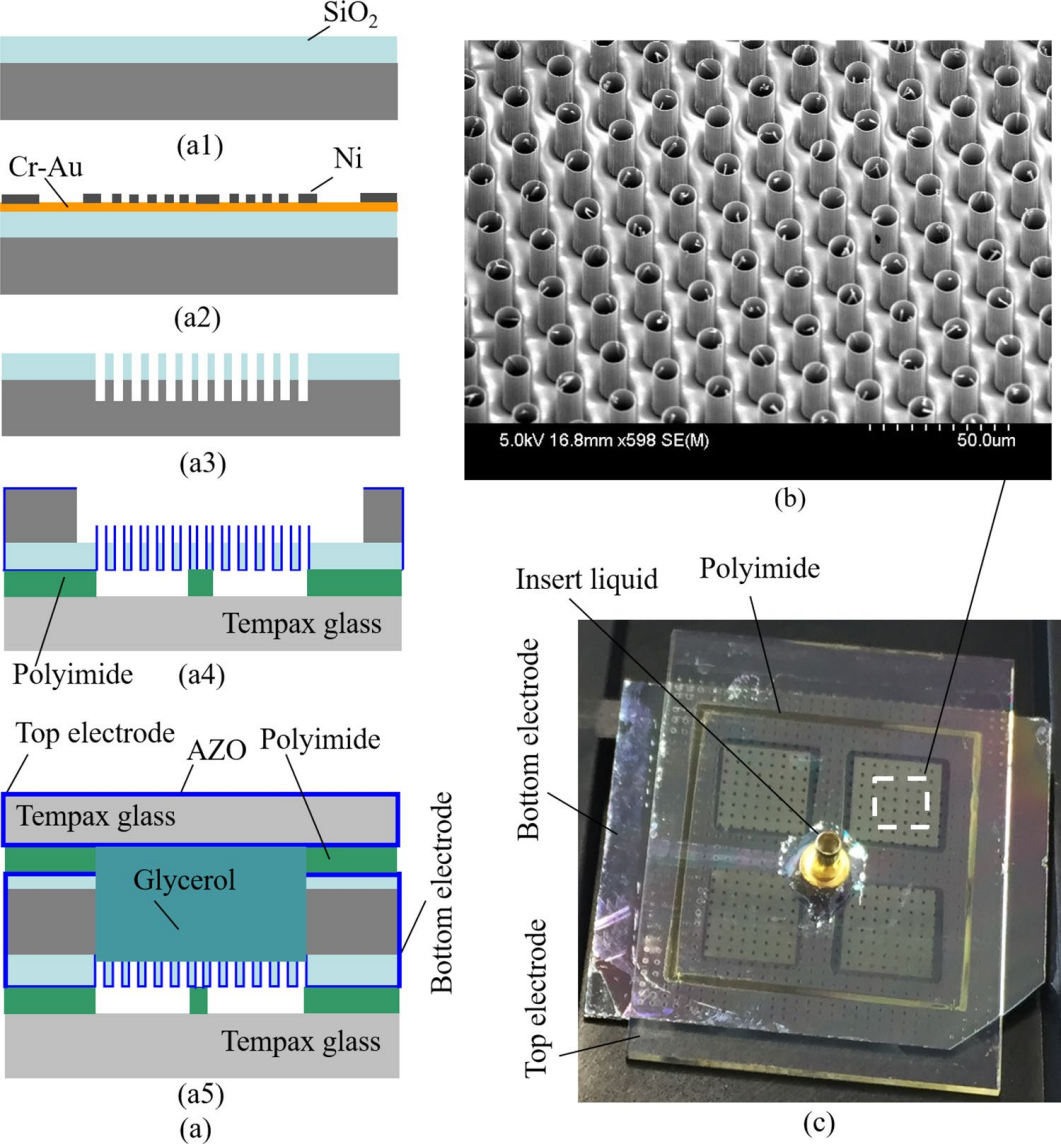
Optical modulator. (**a**) Fabrication process. (**a1**) Thick SiO_2_ deposition on a silicon wafer by PECVD. (**a2**) Ni electroplating. (**a3**) Etched SiO_2_ by reactive ion etching. (**a4**) Transferring process to a glass substrate with polyimide pillars. (**a5**) Bonding with the liquid reservoir. (**b**) Fabricated AZO capillaries. (**c**) Completely fabricated device.

Figure 5a illustrates the fabrication process which starts with a 300 µm-thick silicon wafer. After conventional cleaning, a SiO_2_ layer with a thickness of 15 µm is deposited by plasma-enhanced chemical vapor deposition (PECVD), as shown in Fig. 5a1. Next, 10 nm-thick Cr and 100 nm-thick Au layers as a seed layer for electroplating of Ni are deposited on the SiO_2_ surface by sputtering. Photolithography with a positive photoresist (OFPR-800 200Cp, Tokyo Ohka Kogyo Co. Ltd) is performed on the Au surface of the wafer followed by forming 1 µm-thick pure nickel film with an electroplating method (Fig. 5a2). The OFPR photoresist is then removed with acetone, and the thick SiO_2_ layer and a part of the silicon substrate are etched out by the RIE process. More detailed information on RIE etching of the thick SiO_2_ film can be found in our previous reports^64^. The electroplated nickel and Cr-Au layers are subsequently removed by wet chemical etching (Fig. 5a3). The 50 nm-thick AZO film is deposited by ALD. To enhance the mechanical strength of the SiO_2_ window, a Tempax glass with 10 µm-height polyimide pillar structures is prepared for the transferring process which is performed at 350 °C for 30 min. Then, the backside of the silicon wafer is selectively etched by the deep RIE method until reaching the SiO_2_ layer and exposing AZO structures (Fig. 5a4). Thin AZO layer covered top capillaries is etched out by ion beam milling. Approximately 400 nm-thick Cytop is deposited on the wafer by a dip-coating machine (SDI co. Ltd.). The wafer is then sealed by Tempax glass substrate which contains AZO thin film coating and inlet holes (Fig. 5a5). Finally, the Glycerol liquid is inserted through the inlet hole. Figure 5b shows the SiO_2_ layer and AZO capillary structures after the backside deep RIE of the silicon wafer. Final bonding and inlet hole mounting are shown in the completed device structure, as demonstrated in Fig. 5c.

**Figure 6:**
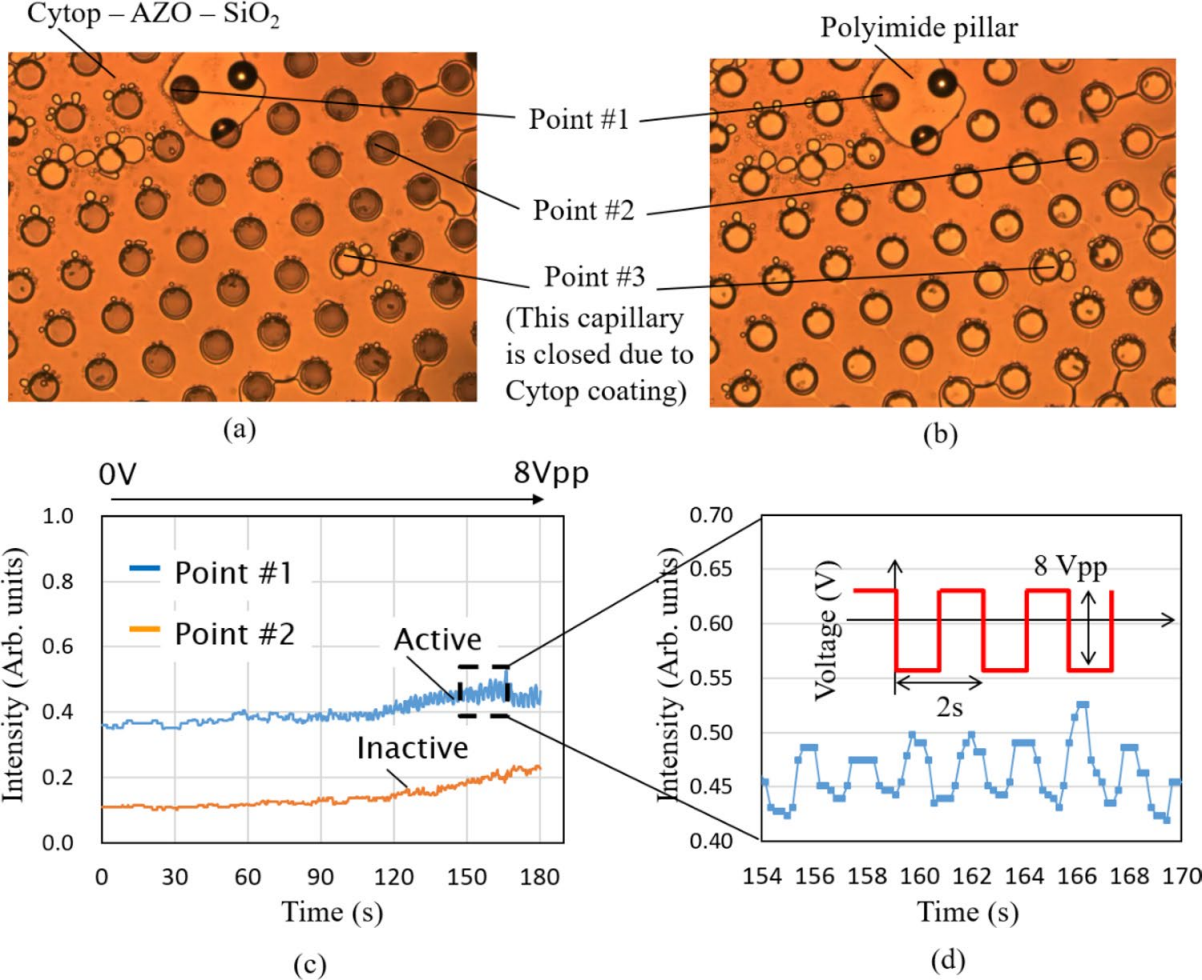
Evaluation results. (**a**) Without voltage supply. (**b**) With voltage supply. (**c**) Quantitative evaluation. (**d**) Liquid oscillation.

The evaluation setup is the same as the previous report^62^. A charge-coupled device (CCD) detector and halogen lamp are used, and on the device a CCD detector is set on the top side, and a halogen lamp is placed at the bottom side via a mirror. The top and bottom electrodes of the fabricated device are connected to the pulse source. Figure 6a, b show the optically captured images without and with a voltage supply, respectively. Point #1 indicates the area of the Polyimide pillar which uses for the transferring process, as mentioned above. Point #2 is the open capillary while Point #3 is the close capillary caused by the Cytop dip-coating. Few capillaries are closed due to the Cytop coating. The possible reason may be owing to the remaining AZO membrane which is not removed completely. With 8 V supply to the electrodes, the center area of the capillaries becomes clear due to the liquid penetration into the capillaries, which reduces the light reflection or scattering at the capillary sidewalls. A qualitative comparison between with and without voltage supply to the electrodes is shown in Fig. 6a, b. To demonstrate the liquid oscillation, the pulse voltages have been applied with a cycle of 2 s. The liquid oscillation has been observed from the light intensity change, as shown in Fig. 6c, d. Although liquid oscillation in capillaries has been achieved, the light intensity variation is still in a small value. It is necessary to produce a smaller and deeper capillary window to obtain higher light modulation toward the production. We believe that optimum versions of this device are one of the promising options for optical focusing which can replace microlens array in the current digital cameras.

### Nanomechanical resonators

Nanomechanical resonators keep an important role in a variety of applications such as high-sensitive sensors^5^, timing and frequency reference^67^, molecular mass detection^68^, and quantum information processing^69^ owing to their advantages including the small size, high-frequency range, and low power consumption. Herein, the fabrication and evaluation of different nanomechanical resonators from a simple structure (a bridged nanobeam structure) to a complex structure (a vertical nanomechanical capacitive resonator) are investigated and demonstrated.

**Figure 7:**
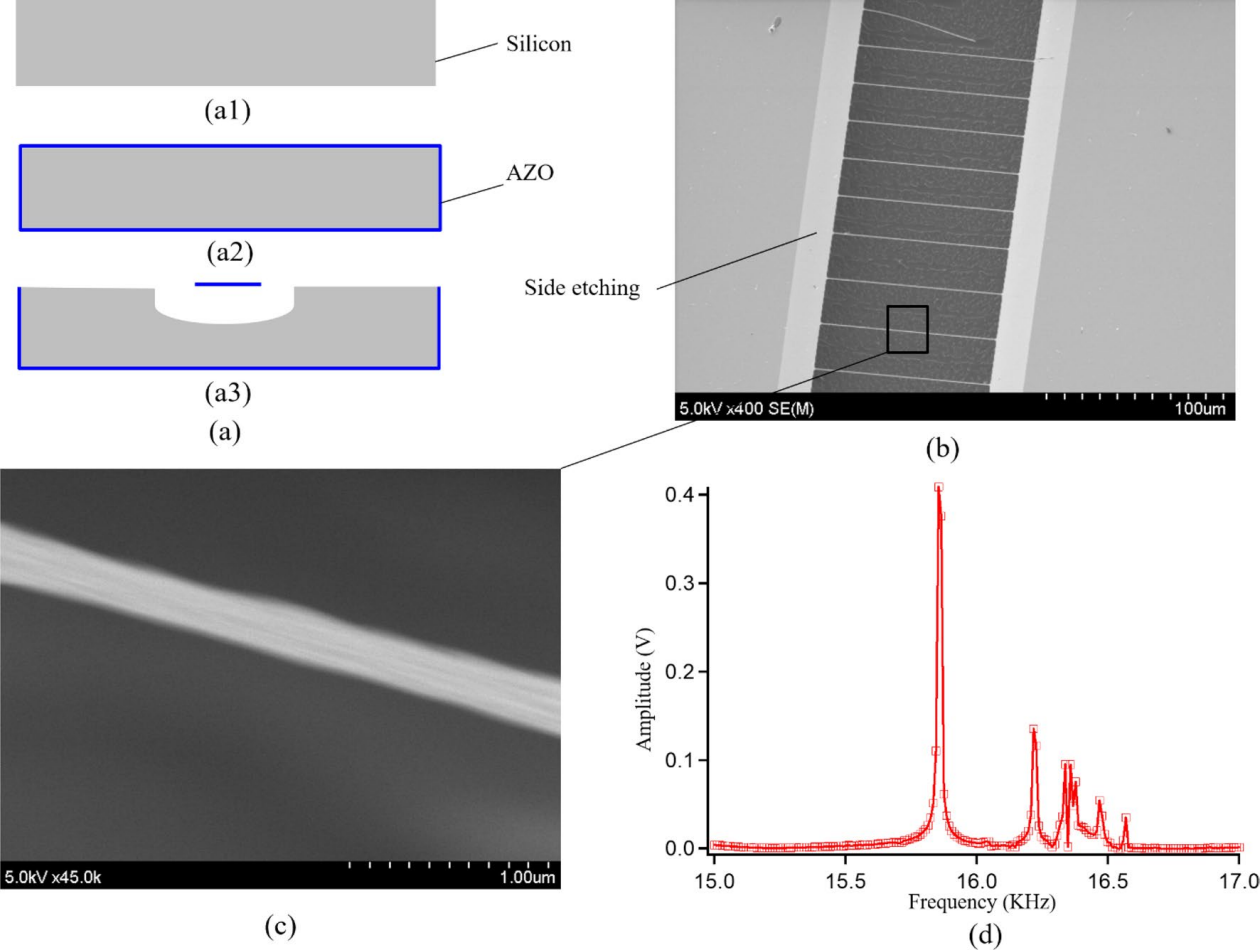
Fixed–fixed nanobeam structure. (**a**) Fabrication process. (**a1**) Silicon wafer. (**a2**) ALD process. (**a3**) Nanobeam structure. (**b**) Fabricated nanobeam structure. (**c**) Close-up image of nanobeam structure. (**d**) Frequency response.

The nanobeam structure is produced by a simple fabrication process, as shown in Fig. 7a. Common polished silicon with a thickness of 300 µm is employed as a substrate (Fig. 7a1). The AZO thin film with a thickness of 50 nm is coated on the entire surface of the wafer, as shown in Fig. 7a2. The AZO thin film is patterned by a diluted hydrofluoric (HF) to form AZO nanobeam structures and silicon underneath the nanobeams are subsequently removed by *SF*_6_ plasma etching, as shown in Fig. 7a3. The over-etching process is performed to ensure the releasing process. Figure 7b shows the successfully fabricated bridged AZO nanobeam structures with a width of approximately 300 nm (Fig. 7c), a length of 100 µm, and a thickness of 50 nm. The fabricated structures are placed in a vacuum chamber and vibrated by employing a PZT actuator. The mechanical resonance is detected by a laser Doppler vibrometer via a lock-in amplifier. More information about the evaluation setup could be found in our previous works^10^. Its resonant frequency is observed at 15.9 kHz and the quality factor is found at 9936, as shown in Fig. 7d. Thus, the AZO thin film material formed by the ALD process poses a high-quality film as a mechanical material that is comparable to silicon material.

Next, the vertical nanomechanical capacitive resonator is presented. A capacitive displacement detection technique is typically employed for monitoring the changing capacitance between the electrodes and the resonant body^70,71^. However, a facing issue for this technique in the nanomechanical capacitive resonator structure is its height at the nanoscale which results in a very small value of the capacitance between electrodes and a resonant body. Thus, it is a great challenge to drive and sense the motion of the capacitive resonators. An increase in the capacitance could improve the performance of the nanomechanical capacitive resonators by means of increasing electromechanical coupling for lower driving voltages and higher sensitivity of the nanomechanical motion. Herein, the vertical nanomechanical resonator has been developed to get overcome the issue.

**Figure 8:**
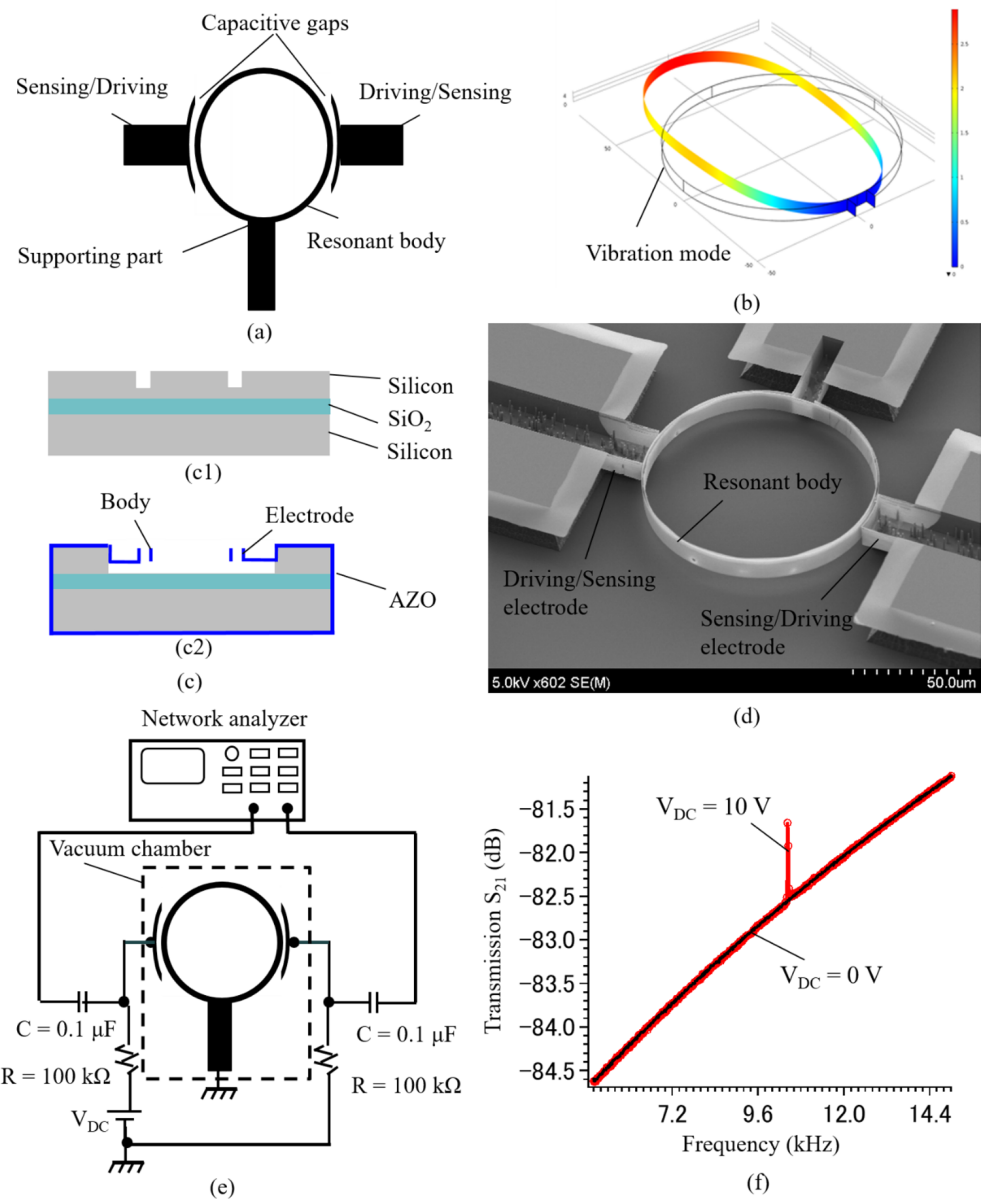
AZO nanomechanical resonator. (**a**) Vertical nanomechanical capacitive resonator. (**b**) Vibration mode by FEM simulation. (**c**) Fabrication process. (**c1**) Silicon pattering. (**c2**) AZO capacitive resonator. (**d**) Fabricated AZO capacitive silicon resonator. (**e**) Evaluation setup. (**f**) Frequency response of the AZO capacitive resonator.

Figure 8a shows the proposed schematic of the AZO vertical nanomechanical resonator which consists of a nanowall hollow cylinder resonant body, driving and sensing electrodes, and narrow capacitive gaps. The resonant body is fixed on the side by a thin supporting part and separated from the electrodes. The working principle of this device is similar to other capacitive silicon resonators presented in our previous works^53^. It can be briefly described as following: the resonant body is excited and vibrated by an electrostatic force that is generated by a combination of *V*_DC_ and *V*_AC_ voltages. The output of the capacitive resonator is obtained by a change of the capacitance between the sensing electrode and the resonant body. Its vibration mode is illustrated by a finite element method (FEM) simulation, as demonstrated in Fig. 8b.

The AZO vertical nanomechanical resonator is also fabricated by a combination of the ALD and deep RIE, as shown in Fig. 8c. An SOI wafer (same as above mentioned) is used as a starting substrate. To form the narrow capacitive gaps and resonator shape, an electron beam lithography in combination with the deep RIE is employed. A similar fabrication process could be found at^53^. Herein, the silicon device layer (15 µm) is just etched out partly with the etching depth of 10 µm, as shown in Fig. 8c1. Next, the AZO thin film with a thickness of 50 nm is coated on the patterned wafer by ALD. The photolithography and selective silicon etching are subsequently conducted. Finally, the suspended AZO hollow disk capacitive resonator can be accomplished (Fig. 8c2). The successfully fabricated device is shown in Fig. 8d. The AZO vertical nanomechanical resonator with a high aspect ratio of 200 for the nanowall in the resonant body (50 nm-width and 10 µm-height) and capacitive gaps of 300 nm has been demonstrated. Figure 8e shows the evaluation setup for a frequency response characterization of the fabricated device. A network analyzer, DC source, vacuum chamber, and several electrical components are employed for this evaluation. More information about the measurement setup for capacitive resonators could be found in our previous works^53^. The frequency response of the fabricated device is shown in Fig. 8e. The resonant peak is found at 10.4 kHz with a quality factor of approximately 500 under measurement conditions of *V*_DC_ = 10 V and *V*_AC_ = 0 dBm, as shown in Fig. 8f. In summary, the vertical nanomechanical AZO capacitive resonator is developed successfully by the use of ALD in combination with sacrificial silicon structures. This demonstrated resonant structure may be helpful for several different applications in biomedical engineering, and life sciences.

## Conclusion

This work not only investigates the deposition and evaluation of the AZO thin film but also demonstrates its novel applications to micro-and nano-devices. 50 nm-thick AZO thin film with high uniformity has been deposited successfully by ALD process and its characteristics are evaluated via SEM, EDX, SAED and HR-TEM. The utilization of the ALD process in combination with sacrificial silicon structures could create high aspect ratio AZO structures. Moreover, three kinds of micro- and nano-devices including micro-hollows with the high aspect ratio nanowall structure, micro- and nano-fluidics (the hollow fluidic resonator and the optical modulator), and nanomechanical resonators (the fixed–fixed nanobeam structure and the vertical nanomechanical capacitive resonator) are fabricated and evaluated. Thus, this development of the AZO thin film deposited by ALD process would open broad applications in micro- and nano-devices required high aspect ratio structures.
